# Analysis of microRNA transcription and post-transcriptional processing by Dicer in the context of CHO cell proliferation

**DOI:** 10.1016/j.jbiotec.2013.12.018

**Published:** 2014-11-20

**Authors:** Matthias Hackl, Vaibhav Jadhav, Gerald Klanert, Michael Karbiener, Marcel Scheideler, Johannes Grillari, Nicole Borth

**Affiliations:** a Department of Biotechnology, BOKU – University of Natural Resources and Life Sciences, Vienna, Austria; b ACIB GmbH, Austrian Centre of Industrial Biotechnology, Graz, Austria; c RNA Biology Group, Institute for Genomics and Bioinformatics, Graz University of Technology, 8010 Graz, Austria

**Keywords:** Chinese hamster ovary cells, MicroRNA, Dicer, Cell engineering, Microarray

## Abstract

CHO cells are the mammalian cell line of choice for recombinant production of therapeutic proteins. However, their low rate of proliferation limits obtainable space-time yields due to inefficient biomass accumulation. We set out to correlate microRNA transcription to cell-specific growth-rate by microarray analysis of 5 CHO suspension cell lines with low to high specific growth rates. Global microRNA expression analysis and Pearson correlation studies showed that mature microRNA transcript levels are predominately up-regulated in a state of fast proliferation (46 positively correlated, 17 negatively correlated). To further validate this observation, the expression of three genes that are central to microRNA biogenesis (Dicer, Drosha and Dgcr8) was analyzed. The expression of Dicer, which mediates the final step in microRNA maturation, was found to be strongly correlated to growth rate. Accordingly, knockdown of Dicer impaired cell growth by reducing growth-correlating microRNA transcripts. Moderate ectopic overexpression of Dicer positively affected cell growth, while strong overexpression impaired growth, presumably due to the concomitant increase of microRNAs that inhibit cell growth. Our data therefore suggest that Dicer dependent microRNAs regulate CHO cell proliferation and that Dicer could serve as a potential surrogate marker for cellular proliferation.

## Highlights

• The expression of Dicer is correlated to growth rate in different CHO cell lines.
• Global perturbation of microRNA levels via DICER knockdown or overexpression directly influences CHO growth behavior.
• This provides strong evidence that microRNAs are key growth regulators in CHO cell lines.

## 1 Introduction

Recombinant expression of therapeutic proteins in Chinese hamster ovary (CHO) cells has a long history (Hacker et al., 2009, Jostock and Knopf, 2012), due to the ease of cultivation of CHO cells in suspension and protein-free media, the availability of tools for clone selection and gene amplification and due to various safety aspects (reviewed by Wurm, 2004). Collaborative effort has recently been put into their characterization in terms of genome (Brinkrolf et al., 2013, Lewis et al., 2013, Xu et al., 2011), cDNA (Becker et al., 2011, Rupp et al., 2014) and non-coding RNA sequencing projects (Hackl et al., 2012b, Hackl et al., 2011, Johnson et al., 2011) as well as characterization of the CHO proteome (Baycin-Hizal et al., 2012, Meleady et al., 2012a) and metabolome (Martínez et al., 2013). These data are essential for understanding and eventually also predicting and adapting CHO cell phenotypes to the requirements of modern bioprocesses.

One approach to increase yields from mammalian bioprocesses is to increase the viable cell number by reducing the rate of apoptosis. Therefore, multiple cell engineering strategies were developed to increase apoptosis resistance of CHO cells by overexpression of endogenous (Han et al., 2011) or evolved anti-apoptotic proteins of the Bcl-family (Majors et al., 2012). Sophisticated transcriptomic, proteomic and metabolomic approaches identified bottlenecks in the energy metabolism of CHO cells that prevent efficient growth and/or protein production (Chong et al., 2010, Doolan et al., 2010). These limitations might be overcome by engineering the expression of single genes, however, the alteration of entire gene networks seems most promising, but at the same time most difficult. In order to meet the challenge of manipulating entire gene networks without burdening the translational machinery of a cell factory, non-coding RNAs, and especially microRNAs (miRNAs) constitute a promising alternative (Hackl et al., 2012a, Jadhav et al., 2013). To this date, miRNAs in CHO cells were identified to regulate growth (Jadhav et al., 2012), stress resistance (Druz et al., 2011) or specific productivity (Barron et al., 2011) by repressing the expression of hundreds of target genes (Meleady et al., 2012b). In fact, across all cell biological disciplines these small (18–24 nt) RNAs have been widely recognized as central regulators of cellular phenotype (Kosik, 2010), with potential applications beyond cell engineering as therapeutic targets (Rooij et al., 2012) or diagnostic markers of disease (Velu et al., 2012). miRNAs are transcribed mostly from RNA Polymerase II promoters in the genome, or excised from intronic regions of mRNA primary transcripts (Carthew and Sontheimer, 2009). These primary miRNA transcripts (pri-miRNAs) consist of a stem-loop structure flanked by single-stranded RNA regions and are subject to two sequential maturation steps: in the nucleus the “microprocessor complex” formed by Drosha and Dgcr8 binds pri-miRNAs and cleaves off a ∼50–80 nt long precursor-miRNA (pre-miRNAs) structure containing the RNA stem-loop (Gregory et al., 2004). Export into the cytoplasm occurs via Exportin-5 and results in the association of pre-miRNAs with Dicer, a ∼230 kDa protein of the helicase family consisting of two RNase-III domains as well as RNA binding, helicase and protein interaction domains (Soifer et al., 2008, Takeshita et al., 2007). Dicer cleavage sets free a ∼22 nt miRNA duplex, from which the guide miRNA is selected and incorporated into a large protein complex called RISC (RNA-induced silencing complex). miRNAs select their targets by imperfect base-pairing to recognition sites present in 3′UTRs or coding regions of messenger RNA (mRNA). The relative position of miRNA:mRNA interaction and the type of Argonaute protein incorporated in the miRNA-RISC decides whether translational repression or mRNA destabilization and degradation will occur (Carthew and Sontheimer, 2009). The imperfect nature of miRNA:target interaction allows single miRNAs to repress the expression of hundreds of different mRNAs, depending on target mRNA availability as well as interaction site accessibility (Arvey et al., 2010), thus attributing miRNAs an important role in the global regulation of gene expression similar to transcription factors (Hobert, 2008).

In addition to the exploration of miRNA function by overexpression, knockdown and target validation studies, studies of miRNA biosynthesis and the regulation of this multistep process have been conducted (Davis-Dusenbery and Hata, 2010, Krol et al., 2010). It is known that the maturation of specific pri-miRNAs by Drosha is dependent on the binding of proteins, for example p53 which induces the biosynthesis of selected growth-suppressive miRNAs (Suzuki et al., 2009). Unlike Drosha activity, which generally requires binding of auxiliary proteins, Dicer is constitutively active which is mirrored in low detectable levels of pre-miRNAs compared to pri-miRNAs or mature miRNAs (Lee et al., 2008). Rather, regulation of miRNA biosynthesis at the Dicer step depends on the inhibition of Dicer activity, or on the de-regulation of Dicer expression, which have been observed during organism development (Rybak et al., 2008), disease progression (Coley et al., 2010, Han et al., 2010) or even in vitro cultivation (Asada et al., 2008, Hwang et al., 2009). As a consequence, mature miRNA levels are subject to change on a global scale under these conditions, thus broadly affecting gene expression.

To our best knowledge, no study has addressed the biological effect of deregulated miRNA biogenesis in CHO cells. Based on miRNA microarray data from five CHO suspension cell lines with slow to high proliferation rates, we observed a global increase in miRNA transcripts along an increase in growth rate. In order to test whether this shift in miRNA transcript levels is assisted or caused by enhanced miRNA transcription or maturation, expression analyses of Dicer, Drosha and Dgcr8 were performed, as well as functional analysis of Dicer by performing loss- and gain-of-function experiments.

## 2 Material and methods

### 2.1 Cell culture

#### 2.1.1 Cell maintenance

Suspension and serum-free adapted CHO-DUKXB-11 cells were grown in DMEM:Ham’ F12 (1:1) supplemented with 4 mM l-glutamine and protein-free additives without growth-factors (CHO-DUKXB-11). All other cell lines were cultivated in CD CHO media (Life Technologies) supplemented with 8 mM l-glutamine (CHO-K1-8 mM and CHO-S) or without (CHO-K1-0 mM) and 1:500 anti-clumping agent (Life Technologies). Recombinant CHO-DUKXB-11 cells expressing an erythropoietin-Fc fusion protein were grown in suspension in CD CHO media with 0.019 μM methotrexate and without l-glutamine supplementation (Taschwer et al., 2011). No defined growth factors such as Insulin or IGF were used as additives in this study.

All cell lines were cultivated in suspension in Erlenmeyer shake flasks in 50 ml volume at 140 rpm in a shaking incubator (Kuhner, Switzerland) in a humidified atmosphere (90%) conditioned with 7% CO_2_.

#### 2.1.2 Generation of stable Dicer overexpressing pools

CHO-DUKXB-11 host cells (10^7^ cells in total) were transfected by nucleofection (LONZA) with 10 μg of recombinant human Dicer plasmid (Genecopoeia, GC-H0470) containing the open reading frame of human Dicer (NM_030621.2 and NP_085124.2) under a CMV promoter and neomycin resistance gene. Post-transfection, cells were seeded at a concentration of 3.0 × 10^5^ cells/ml in 30 ml media and maintained at 37 °C with humidified air, 7% CO_2_, and constant shaking at 140 rpm for 24 h. At this point, selection media containing 800 μg/ml G418 (Invivogen, San Diego, USA) was added, and cells were transferred to a 96 well plate at a concentration of 10,000 cells/well. Throughout selection, media was replaced every 3–4 days, and wells with growing cells were expanded to 12-well plates after 4 weeks of selection. At this stage individual wells containing stable growing CHO pools were tested for human Dicer1 incorporation and expression by PCR amplification from genomic DNA (gDNA) and copied DNA (cDNA) using specific primers (Supporting Table S1) and Western blot as described below (2.5).

#### 2.1.3 siRNA mediated knockdown of Dicer

For targeted knockdown of Dicer expression in CHO cells, two 21 nt long siRNAs were designed based on the NCBI reference sequence NM_001244269.1: siRNA#1 target site: GAGTGGTAGCTCTCATTTGCT; siRNA#2 target site: TAACCTGGAGCGGCTTGAGAT. All siRNAs were custom synthesized at 25 nm scale (Qiagen, Germany). For transfection, both siRNAs were pooled at equimolar concentration. As control, a non-targeting RNA duplex was designed (GUGUAACACGUCUAUACGCCCA) and custom synthesized (Biomers, Germany). Small RNAs were transfected at 30 nM concentration in three replicates in 6-well plate format. ScreenfectA (Incella, Germany) was used for lipid/RNA complex formation according to the provided protocol. Cells were seeded at 3.5 × 10^5^ cells/ml in 2.5 ml, before complexed siRNAs were added to each well. Cultivation was performed at 37 °C in humidified air with 7% CO_2_ and constant shaking at 60 rpm. After 72 h cells were harvested for RNA isolation and cell density/viability measurements.

### 2.2 RNA Isolation

Isolation of total RNA was performed using phenol–chloroform extraction from Trizol lysed CHO cell pellets. In brief, CHO suspension cells were lysed in 1 ml TRI reagent (Sigma–Aldrich) and stored at −80 °C or processed immediately. Adherent CHO cell lines were detached from the surface by trypsinization, PBS-washed and lysed in 1 ml TRI reagent. RNA extraction using chloroform and purification were performed as described previously (Hackl et al., 2011). RNA pellets were resuspended in nuclease-free water (Life Technologies) and concentrations and purity were analyzed through absorption at 230, 260, and 280 nm using a NanoDrop spectrophotometer (Thermo Scientific).

### 2.3 Determination of RNA quality and small RNA concentration

In order to assess total RNA quality and the fraction of small RNAs and microRNAs, total RNA was diluted to a concentration of 100 ng/μl. Total RNA quality was estimated on a Bioanalyzer 2100 instrument using the RNA 6000 Nano Kit. SmallRNA and microRNA concentrations were measured from the same RNA aliquots using the small RNA Series II Kit according to the instructions by the manufacturer (Agilent Technologies, Santa Clara, USA).

### 2.4 cDNA synthesis and PCR and real-time quantitative PCR

Total RNA in various amounts ranging between 200 ng and 1 μg was used for cDNA synthesis using a M-MuLV RNase H+ reverse transcriptase supplied with the Dynamo Kit (Thermo Scientific). cDNA was diluted in nuclease-free water depending on the initial input of total RNA and directly used for end-point PCR as well as real-time quantitative PCR (qPCR). PCR analysis of human Dicer expression was performed using a Taq polymerase provided with the Phusion high-fidelity polymerase kit (Thermo Scientific) with 35 cycles of denaturation (95 °C, 15 s), annealing (58 °C, 20 s) and extension (72 °C, 20 s).

For quantitation of mRNA expression, specific qPCR primers that overlap exon–exon junctions or are separated by at least one intron, were designed for beta-Actin (Actb), human and Chinese hamster Dicer, Drosha, and Dgcr8 and are provided in Supporting Table S1. Primer specificity was tested by melting curve analysis. Standards for copy number determination were prepared by purification of PCR products and dilution to 10^8^–10^3^ copies/μl and included in each run. Quantitative PCRs were run in quadruplicates on a Rotorgene Q (Qiagen), using SYBR green fluorescent dye and a hot-start polymerase supplied with the SensiMix mastermix (Bioline) with 40 cycles of denaturation (95 °C, 15 s), annealing (60 °C, 15 s) and elongation (72 °C, 15 s). SYBR Green fluorescence was acquired at 72 °C and 80 °C, and chosen for detection depending on the base of the melting peak.

### 2.5 Analysis of microRNA transcription

#### 2.5.1 MicroRNA microarray hybridization

Cross-species microRNA microarray experiments were run as described previously (Hernández Bort et al., 2012). In brief, epoxy-coated Nexterion glass slides were spotted using the miRBase version 16.0 locked nucleic acid (LNA) probe set consisting of 2367 probes against human, mouse and rat miRNAs in 8 replicates. For hybridization, 800 ng total RNA extracts from two biological replicates of each cell line from exponential growth phase were hybridized against a common reference pool RNA from all samples. End-labeling of miRNAs was performed using the Exiqon Power Labeling Kit (Exiqon, Denmark) together with synthetic spike-in controls according to the instructions by the manufacturer. Slides were hybridized over night at 56 °C in a Tecan HS 400 hybridization station, followed by automated washing and drying with nitrogen (Tecan, Austria). Immediately after drying, arrays were scanned using the Roche Nimblegen MS200 scanner (Roche, Germany) at 10 μM resolution and auto-gain settings.

#### 2.5.2 MicroRNA microarray data analysis

Feature extraction from high-resolution tiff-images was performed using GenePix software (Molecular Devices, Sunnyvale, CA). Background correction, normalization and statistical analysis were performed as previously described (Hackl et al., 2010), using the LIMMA package under R/Bioconductor (Smyth, 2004). *Normexp* background correction and *Global Loess* normalization were performed and log_2_-fold changes of miRNAs for each sample were calculated against the common reference sample and served as relative expression value for each miRNA. Pearson correlation was performed to test for positive or negative correlation of miRNA expression with specific growth rate. Normalized as well as raw microarray data have been submitted to Gene Expression Omnibus (http://www.ncbi.nlm.nih.gov/geo/) and can freely be loaded and reanalyzed using the accession number GSE52994.

#### 2.5.3 MicroRNA qPCR analysis

In order to quantify mature miRNA transcript levels as well as precursor miRNA levels, the miScript kit was used (Qiagen, Germany). Reverse transcription (RT) was performed using 300–400 ng of total RNA and “HiFlex” RT Buffer, which allows detection of both microRNA and messengerRNA. Temperature settings were chosen according to the suppliers recommendations (37 °C for 1 h, 95 °C for 5 min). cDNA was diluted 1:4 in nuclease-free water and qPCRs were run in quadruplicates using the miScript SYBR Green Kit (Qiagen, Germany) on the Rotorgene Q instrument (Qiagen, Germany): 95 °C → 15 min, 40 cycles of 94 °C → 15 s, 55 °C → 30 s, 70 °C → 30 s. SYBR Green fluorescence was measured at 70 °C and 80 °C. Commercial primer assays (Qiagen, Germany) were used for mature miRNA quantification. In-house designed primer assays were used for precursor-miRNA quantification (primer sequences are listed in Supporting Table 1).

### 2.6 Western blot

Protein lysates were prepared by cold lysis of 5 × 10^6^ cells in 1× RIPA buffer for 15 min and centrifugation at 12,000 × *g* and 4 °C for 10 min. Total protein concentration was measured by BCA assay (Pierce), and equal amounts of protein were denatured in 1× LDS buffer with 1× reducing agent (Life Technologies) at 70 °C for 10 min. Samples were separated on 4–15% gradient SDS-PAGE gels (Biorad), blotted onto PVDF membrane, blocked with 3% dry milk in 1× PBS/0.1% Tween 20 (Sigma–Aldrich) and incubated with mouse anti-beta-Actin IgG (1:20,000, Sigma) or rabbit anti-Dicer IgG (1:1000, Sigma–Aldrich) at 4 °C over night. Detection was performed with the IR-Dye system on an Odyssey scanner (Licor) after incubation with anti-mouse (1:10,000) or anti-rabbit (1:5000) secondary antibodies for 60 min at room temperature. Western blot images were analyzed with ImageJ software (Abramoff et al., 2004).

## 3 Results

### 3.1 miRNA transcription in protein-free adapted suspension cell lines with low, medium, and high proliferation rates

**Fig. 1:**
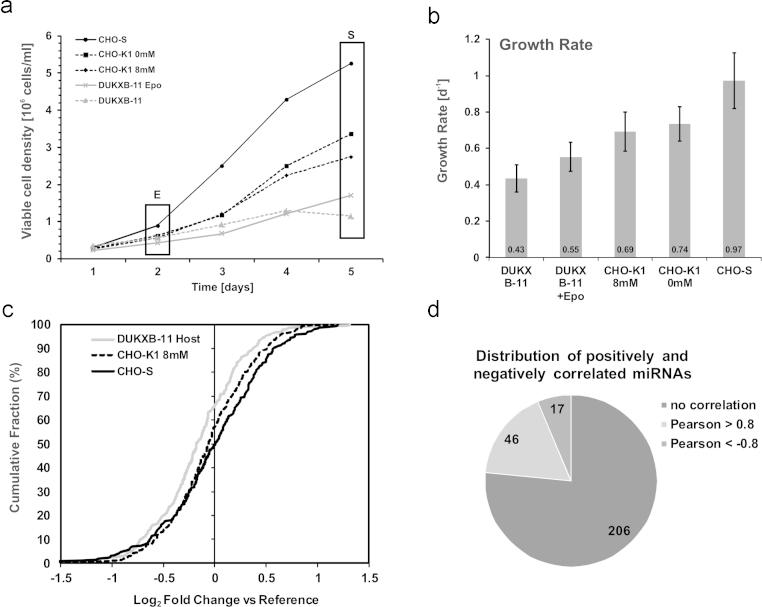
Global microRNA transcription correlates with growth-rate in protein-free and suspension adapted CHO cell lines. (a) Five CHO cell lines were selected for cultivation in chemically defined media. Two individual batch cultivations were performed and samples were harvested during exponential and stationary growth phase. (b) For each cell line, the specific growth rate was calculated between day 1 and day 3. (c) RNA from exponential growth phase was used for miRNA microarray hybridization. Log_2_ transformed fold changes in miRNA expression between each cell line and the common reference pool are shown (average from *n* = 2 per group). Data from DUKXB-11 Epo and CHO-K1 0 mM are not shown. (d) The relationship between miRNA transcript level and specific growth rate was analyzed by Pearson correlation. miRNAs with Pearson correlation coefficients (PCC) greater 0.8 or smaller −0.8 were filtered. Distribution of miRNAs with positive (46), negative (17) or no correlation (206) is given.

To investigate the relationship between CHO cell proliferation rate and miRNA transcription in detail, a panel of 5 CHO cell lines that were previously adapted to serum-free growth in suspension were selected and batch cultivations were performed in duplicate in the same chemically defined media without the addition of growth-factors (Fig. 1a). The cell-specific growth rates (*μ*) that were achieved during exponential growth phase in batch cultivations were found to be lowest (0.43 d^−1^) in case of DUKXB-11 host cells and a derived recombinant cell line expressing an Epo-Fc fusion protein (DUKXB-11 Epo, 0.55 d^−1^). Medium *μ* was achieved by CHO-K1 cell lines cultivated in the presence (CHO-K1 8 mM, 0.69 d^−1^) or absence of l-glutamine (CHO-K1 0 mM, 0.74 d^−1^) as described previously (Bort et al., 2010). The highest specific growth rate was achieved by CHO-S cells (0.97 d^−1^). Fig. 1b gives an overview of the average growth rates observed in three individual batch cultivations. Total RNA was isolated during exponential growth phase on day 2 and stationary growth phase on day 5. Analysis of mature miRNA levels was performed only during exponential growth phase using a previously established microarray platform (Hackl et al., 2010, Hernández Bort et al., 2012). A total of 270 miRNA probes gave signals that were significantly above the background. For these miRNAs log_2_-transformed fold changes (LFC) were calculated against the common reference RNA sample and treated as relative expression values. LFC-values were ranked from low to high and plotted for three cell lines (CHO-DUKXB11, *μ* = 0.43 d^−1^; CHO-K1, *μ* = 0.69 d^−1^; CHO-S, *μ* = 0.97 d^−1^) against the cumulative fraction (Fig. 1c). The results show an increase in miRNA transcription from the slow to fast proliferating CHO cells, which was confirmed by qPCR for selected miRNAs on the level of precursor and mature transcripts (Supporting Fig. 1). Pearson correlation coefficients (PCC) of growth rate and mature miRNA expression were calculated, and miRNAs with stringent PCC values greater 0.8 or below −0.8 were regarded as positively or negatively correlated, respectively. This resulted in a total number of 63 growth-correlating miRNAs, of which 46 (73%) exhibited a positive correlation (Fig. 1d).

### 3.2 Expression of Dicer, but not Drosha or Dgcr8 correlates well with cell-specific growth rate of CHO cell lines

**Fig. 2:**
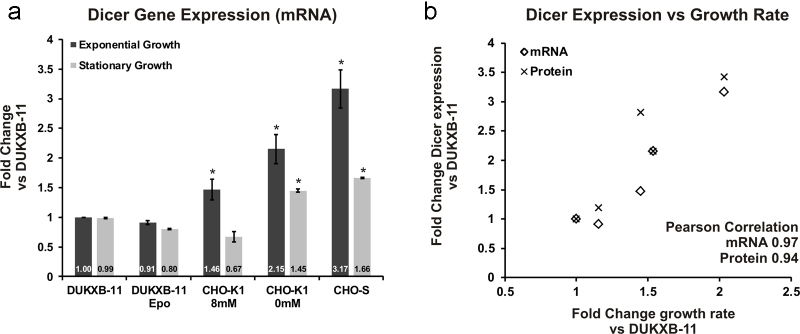
Dicer expression correlates with growth-rate in protein-free and suspension adapted CHO cell lines. (a) qPCR analysis of Dicer transcript levels at two time-points during batch cultivation is shown (dark, exponential growth phase; bright, stationary growth phase). Fold changes are given relative to Dicer levels in DUKXB-11 host cells (*n* = 3 per group, Actb used as reference gene, error bars represent s.d. of mean). Significance tests were performed using pairwise Student’ *T*-test between DUKXB-11 and each cell line. Significant differences (*p* < 0.05) are marked with an asterisk (*). (b) Scatter plot depicting the relationship between specific growth rate and Dicer expression on mRNA (rectangle) and protein (cross) level. Shown are fold changes in expression relative to DUKXB-11 host cells.

In order to test whether increased post-transcriptional processing of miRNAs by Dicer could mediate this effect, Dicer expression was analyzed by qPCR during exponential growth phase, as well as stationary growth phase. Indeed, we observed enhanced expression in fast proliferating cells during exponential phase (Fig. 2a). However, on day 5 when proliferation has decreased due to nutrient consumption and accumulation of toxic metabolites, the difference in Dicer expression was attenuated (Fig. 2b), which is in line with the earlier report of predominant miRNA down-regulation during stationary growth phase (Hernández Bort et al., 2012). Dicer up-regulation during exponential growth phase was further evaluated by immunoblot analysis (Supporting Fig. 2a), which confirmed the strong correlation of Dicer expression and specific growth rate of 5 CHO cell lines (PCC_mRNA_ = 0.97, PCC_protein_ = 0.93, see Fig. 2b). Analogous correlation analyses for Drosha and Dgcr8 expression did not show any significant correlation (Supporting Fig. 2b and c).

These results demonstrated that specific growth rate of CHO cell lines positively correlates with a large fraction of transcribed miRNAs as well as post-transcriptional processing by Dicer. In order to investigate more closely the effect of Dicer expression on CHO cell phenotype, and especially whether the de-regulation of Dicer directly impacts cell proliferation, we conducted loss- and gain-of-function experiments by siRNA-mediated knockdown and ectopic overexpression of Dicer, respectively.

### 3.3 Knockdown of Dicer and consequently miRNA maturation impairs growth of CHO cell lines

**Fig. 3:**
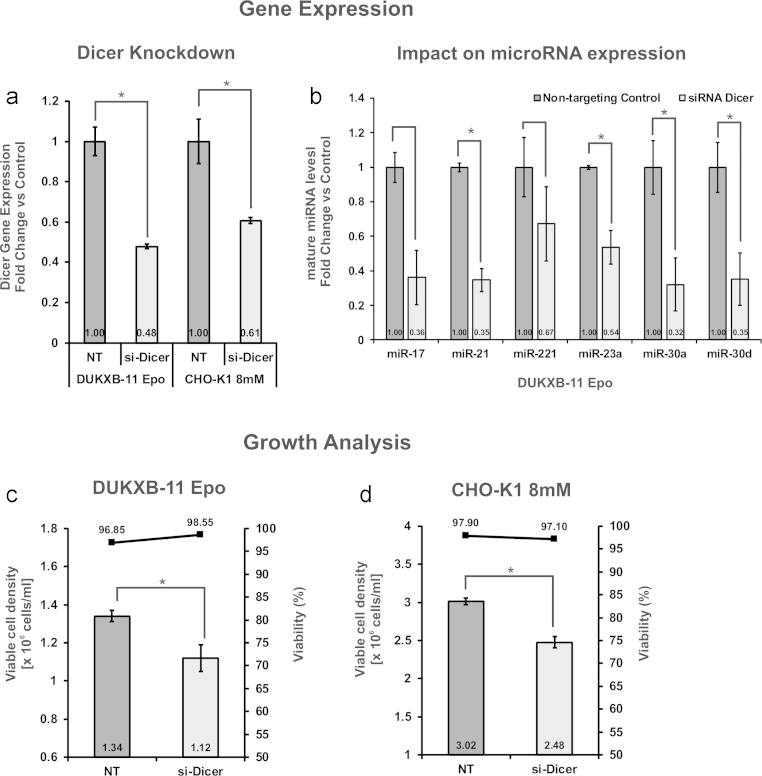
Targeted transient knockdown of Dicer expression using siRNA. Transfection of 30 nM siRNAs targeting Dicer and non-targeting control (NT) was performed in CHO-K1 8 mM and DUKXB-11 Epo cells. Cells were analyzed 72 h post-transfection: Dicer transcript levels (a) were analyzed by qPCR (*n* = 3, Actb used as reference gene, error bars represent s.d. of mean). The impact of Dicer down-regulation on mature miRNA levels was analyzed in DUKXB-11 Epo cells by qPCR (*n* = 3, Actb used as reference gene, error bars represent s.d. of mean). Effect of Dicer knockdown on viable cell density (VCD) and viability of DUKXB-11 Epo (c) and CHO-K1 8 mM cells (d) is shown after 72 h (*n* = 3, error bars represent s.d. of mean). Significance tests were performed using pairwise Student’ *T*-test between non-targeting control (NT) and transfected samples (siRNA). Significant differences (*p* < 0.05) are marked with an asterisk (*).

First, we designed two siRNAs directed against two positions in the coding region of Dicer, which were separated by 1850 nucleotides. Two of the characterized cell lines (DUKXB-11 Epo and CHO-K1 8 mM) with medium proliferation rates were transfected using a recently optimized RNA transfection strategy for CHO cell lines (Fischer et al., 2013) and analyzed 72 h later. This time-point was chosen for analysis, since miRNA half-life is known to range between 24 h and 48 h for most miRNAs (Gantier et al., 2011). Knockdown of Dicer to 60% and 50% residual expression on mRNA level was achieved for both cell lines (Fig. 3a), which resulted in a similar reduction of the levels of 6 selected miRNAs (Fig. 3b). In terms of growth behavior, a significant reduction of viable cell densities by 20% could be observed (Fig. 3c and d), without negatively affecting cell viability. These data suggest that down-regulation of miRNA maturation due to reduced post-transcriptional processing by Dicer limits the proliferation rate of CHO cells.

### 3.4 Ectopic overexpression of Dicer can improve growth of CHO cell lines

**Fig. 4:**
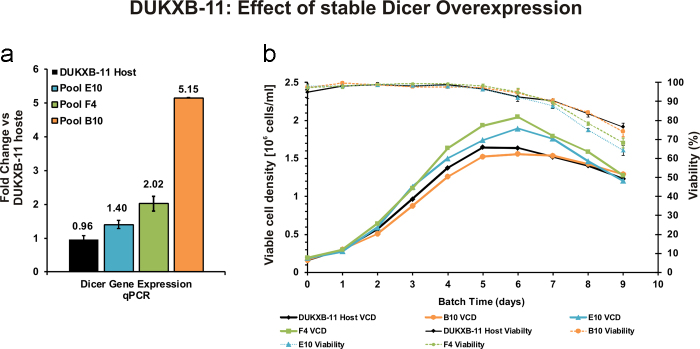
Engineering Dicer expression in CHO DUKXB-11 cells affects growth behavior. (a) Overexpression of Dicer in three stable bulk cell lines compared to DUKXB-11 host cells was analyzed on day 2 during three individual batch cultivations by qPCR (*n* = 3, Actb used as reference gene, error bars represent s.d. of mean). (b) Three independent batch cultivations of all cell lines were performed. Viable cell densities (VCD) and viability were analyzed every 24 h.

**Fig. 5:**
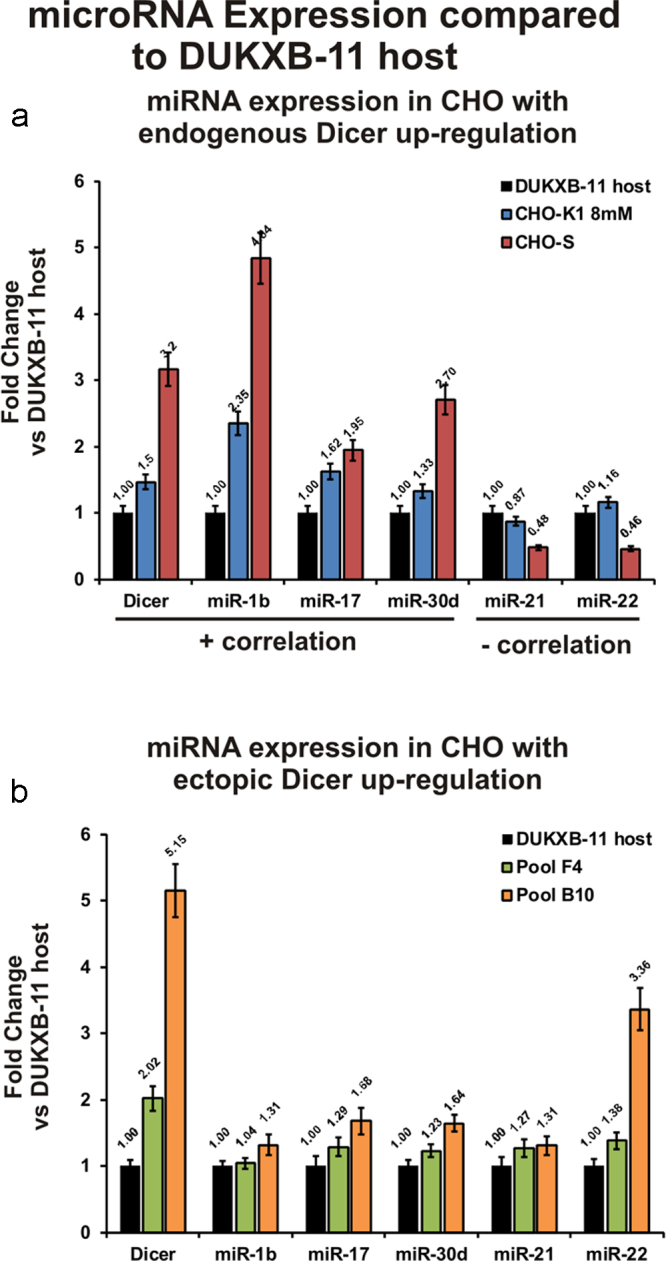
Comparison of miRNA transcription in cell lines with endogenous and ectopic up-regulation of Dicer. DUKXB-11 host cells were chosen as reference for the comparison of miRNA expression in response to endogenous (a) and ectopic (b) Dicer up-regulation. (a) Microarray fold changes for 5 selected miRNAs (three positively and two negatively correlated to growth rate) and qPCR fold change of Dicer expression in CHO-K1 and CHO-S cells relative to DUKXB-11 cells. (b) qPCR fold changes for Dicer and the same miRNAs in two pool cell lines exhibiting moderate and strong ectopic overexpression of human Dicer (*n* = 3, Actb used as reference gene, error bars represent s.d. of mean).

In order to test whether an up-regulation of miRNA maturation by overexpression of Dicer can enhance cell proliferation, we transfected recombinant human endoribonuclease Dicer1 (NP_085124.2), which is 94% homologous to Dicer1 of CHO-K1, into DUKXB-11 host cells, as these cells exhibited the slowest proliferation rate of 0.5 d^−1^. Stable bulk transfected cells were selected for several weeks and screened for human Dicer1 expression by PCR using a primer-pair specific to human Dicer. In order to estimate the overall expression of Dicer in these cells, a primer-pair capable of binding both human and Chinese hamster Dicer was designed (Supp. Tab. S1), and used for qPCR screening: three recombinant cell lines with 1.4-fold (E10), 2.0-fold (F4), and 5.1-fold (B10) increase in Dicer1 expression relative to the host cell line were selected for further characterization (Fig. 4a). Therefore, three independent batch cultivations were inoculated in shake flasks at a viable cell concentration of 1.5 × 10^5^ cells/ml, and grown until viability dropped below 70% at day 9 (Fig. 4b). For E10 and F4, a moderate increase in maximum growth rate (E10, 16.8%; F4, 26.6%) and cumulative cell days (E10, 10.5%; F4, 18.4%) was observed compared to untransfected DUKXB-11 cells (Table 1). This effect also resulted in a 24 h earlier decrease of viability below the 80% threshold (Fig. 4b). Interestingly, the stable pool with highest overexpression of Dicer (B10) showed a decrease in growth performance compared to the host cell line (Tab. 2, Fig. 4b). In order to assess whether Dicer overexpression resulted in an induction of mature miRNA levels, we performed RT-qPCR analysis of 5 miRNAs that were positively (miR-1b, miR-17, miR-30a) or negatively (miR-21, miR-22) correlated to growth rate in our microarray analysis (Fig. 5a). A comparison of miRNA levels between cell lines with significant ectopic overexpression of Dicer (F4, B10) and endogenous up-regulation (CHO-K1, CHO-S) relative to DUKXB-11 host cells is shown in Fig. 5: it was found that (i) ectopic overexpression of Dicer only slightly increases the levels of three selected mature miRNA in CHO cells (Fig. 5b) when compared to the up-regulation observed between fast and slow growing cell lines (Fig. 5a) and (ii) that miRNAs with negative correlation to growth rate (miR-21, miR-22) were also upregulated.

**Table 1:** Growth characteristics of Dicer1 overexpressing CHO cell lines.

	DUKXB-11 HOST	POOL Dicer+ E10	POOL Dicer+ F4	POOL Dicer+ B10
Average *μ*_0–3_ [d^−1^] (%)	0.61 ± 0.01 (100)	0.60 ± 0.06 (98.7)	0.59 ± 0.03 (96.7)	0.58 ± 0.11 (96.1)
Maximum *μ*_1–2_ [d^−1^] (%)	0.65 ± 0.05 (100)	0.75 ± 0.05 (116.8)	0.82 ± 0.04 (126.6^*^)	0.52 ± 0.02 (81.1^*^)
Average IVC_8_ [10^6^ cells days] (%)	7.25 ± 0.65 (100)	8.02 ± 0.34 (110.5)	8.59 ± 0.45 (118.4^*^)	8.30 ± 0.30 (95.2)

^*^ Student’ *T*-test: *p* < 0.05.

Together, these data suggest that enhanced expression of Dicer in fast growing CHO cell lines is a response to increased microRNA transcription rather than the underlying cause of miRNA up-regulation. Nevertheless, moderate overexpression of Dicer does enhance growth performance by 15–20%, presumably due to up-regulation of growth-enhancing miRNAs. However, strong overexpression of Dicer negatively impacts growth behavior as it does not differentiate between specific growth promoting and growth inhibiting microRNAs. Therefore Dicer may be regarded as a surrogate marker for specific growth rate in CHO cells, but does not constitute a promising target for engineering the growth of CHO cell lines.

## 4 Discussion

This study addresses the importance of miRNA regulation in the context of CHO cell proliferation. It was found that ∼75% of mature miRNA transcripts that correlate with cell-specific growth rate across several distinct CHO cell lines, are up-regulated. A similar observation was made in 2012 when Clarke et al. reported 35 positively and only 16 negatively correlated miRNAs when looking at subclones of a single CHO cell line (Clarke et al., 2012).

We therefore raised the question as to how far miRNA processing by Dicer, Drosha and Dgcr8 is relevant for this effect. We found that Dicer mRNA and protein levels – in contrast to Drosha and Dgcr8 levels – positively correlate to cell-specific growth rate during exponential growth phase. However, upon growth arrest during stationary growth phase Dicer is overall downregulated and the difference in Dicer levels between fast and slow growing cell lines is insignificant.

Other studies have reported up-regulation of the entire miRNA protein machinery consisting of Argonaute, Dicer and Drosha along tumor progression – and thus faster growth rates – of serous ovarian carcinoma cells (Vaksman et al., 2012). Furthermore, in endothelial cells the removal of serum was shown to increase cellular sensitivity to apoptosis via the down-regulation of Dicer expression (Asada et al., 2008).

In order to test whether Dicer expression is causally related to growth rate, transient down-regulation of Dicer expression, and in consequence miRNA maturation was performed and indeed significantly decreased the growth rate of CHO cells. To further confirm this relationship, we investigated whether an increase in miRNA maturation by ectopic overexpression of Dicer could improve growth. Therefore, three independent stable pools with Dicer overexpression levels between 1.5 and 5-fold were generated. In batch cultivations these three cell lines show that moderate overexpression of Dicer indeed enhances cell proliferation slightly (∼20%), while more than 5-fold overexpression negatively affected growth performance. In order to investigate the effect of Dicer overexpression, qPCR analysis of selected miRNAs was performed. We observed that ectopic up-regulation of Dicer moderately increased the levels of miRNAs with positive correlation to growth. However, the degree of up-regulation was well below the induction observed for the same miRNAs between fast and slow growing cell lines. In addition, 5-fold induction of Dicer expression also resulted in significant up-regulation of mature miRNAs with negative correlation to growth. This could explain the inhibitory effect of strong Dicer overexpression on growth, and indicates that Dicer is not an ideal engineering target.

Overall it seems that up-regulation of specific miRNAs supports high proliferation rates in CHO cell lines. Simultaneous up-regulation of Dicer seems to be necessary to allow rapid maturation of pre-miRNAs into mature miRNAs, but does itself not mediate growth stimulation. The weaker induction of Drosha and Dgrcr8 could be due to the fact that miRNAs derived from intronic regions can bypass Drosha/Dgcr8 cleavage (Ruby et al., 2007). Therefore, this work establishes Dicer as a potential surrogate marker for growth rate in CHO cells, but not as a promising target for engineering proliferation. For this purpose, it will be worthwhile to test the biological function of those miRNAs exhibiting strong negative or positive correlation to growth rate, such as miR-7 or miR-17, for which respective data already exists (Barron et al., 2011, Jadhav et al., 2012).

## Conflict of interest

The authors declare no conflicts of interest.
