# Supplementary material for: Analysis of microRNA transcription and post-transcriptional processing by Dicer in the context of CHO cell proliferation
Source: J Biotechnol. 2014 Nov 20;190:76–84. doi: 10.1016/j.jbiotec.2013.12.018 (PMC4247382; doi:10.1016/j.jbiotec.2013.12.018)
Supplement: Supplementary file 1 [file mmc1.doc]

**Supporting Table S1: PCR and qPCR primer**

| **Primer Target** | **Species** | **Primer Orientation** | **Sequence (5' - 3')** | ***Tm* (°C)** | **Length (bp)** | **PCR/**  **rt-qPCR** |
| --- | --- | --- | --- | --- | --- | --- |
| ACTB | Cricetulus griseus | Forward | TACGTGGGTGACGAGGCCCA | 59.5 | 387 | rt-qPCR |
|  |  | Reverse | AGCCAGGTCCAGACGCAGGA | 59.5 |  |  |
| Dicer | Homo sapiens | Forward | GGAAGAGCAAGGGCACCCATCTC | 59.3 | 150 | PCR |
|  |  | Reverse | GTCAGTCTCACCAGTATCAACCGAC | 56.9 |  |  |
| Dicer | Homo sapiens  Cricetulus griseus | Forward | GTCAACTCTGCAAACCAGGTTGC | 57.1 | 437 | rt-qPCR |
|  |  | Reverse | CTGTCTAAGACCACCAGGTCAGT | 55.3 |  |  |
| Drosha | Cricetulus griseus | Forward | CTCGGCCTCCATGGGAGCCT | 60.0 | 174 | rt-qPCR |
|  |  | Reverse | CATGCAGCCGGTCAGGGTGG | 60.0 |  |  |
| Dgcr8 | Cricetulus griseus | Forward | TGCGCATGTATGGCCGTGAGA | 58.9 | 307 | rt-qPCR |
|  |  | Reverse | GGCCTGGTGCTGCAGTGATGTGT | 61.8 |  |  |
| pre-mir-17 | Cricetulus griseus | Forward | CAAAGTGCTTACAGTGCAGGTAGTG | 56.7 | 80 | rt-qPCR |
|  |  | Reverse | CCACAAGTGCCTGCACTGCAGT | 65.5 |  |  |
| pre-mir-23a | Cricetulus griseus | Forward | GGGGTTCCTGGGGATGGGATTTG | 67.6 | 76 | rt-qPCR |
|  |  | Reverse | GGAAATCCCTGGCAATGTGAT | 60.7 |  |  |
| pre-mir-30a | Cricetulus griseus | Forward | TGTAAACATCCTCGACTGGAAGCTG | 60.3 | 82 | rt-qPCR |
|  |  | Reverse | GCTGCAAACATCCGACTGAAAG | 61.8 |  |  |
| pre-mir-185 | Cricetulus griseus | Forward | TGGAGAGAAAGGCAGTTCCTGA | 63.5 | 74 | rt-qPCR |
|  |  | Reverse | ACCAGAGGAAAGCCAGCCCCT | 67.6 |  |  |


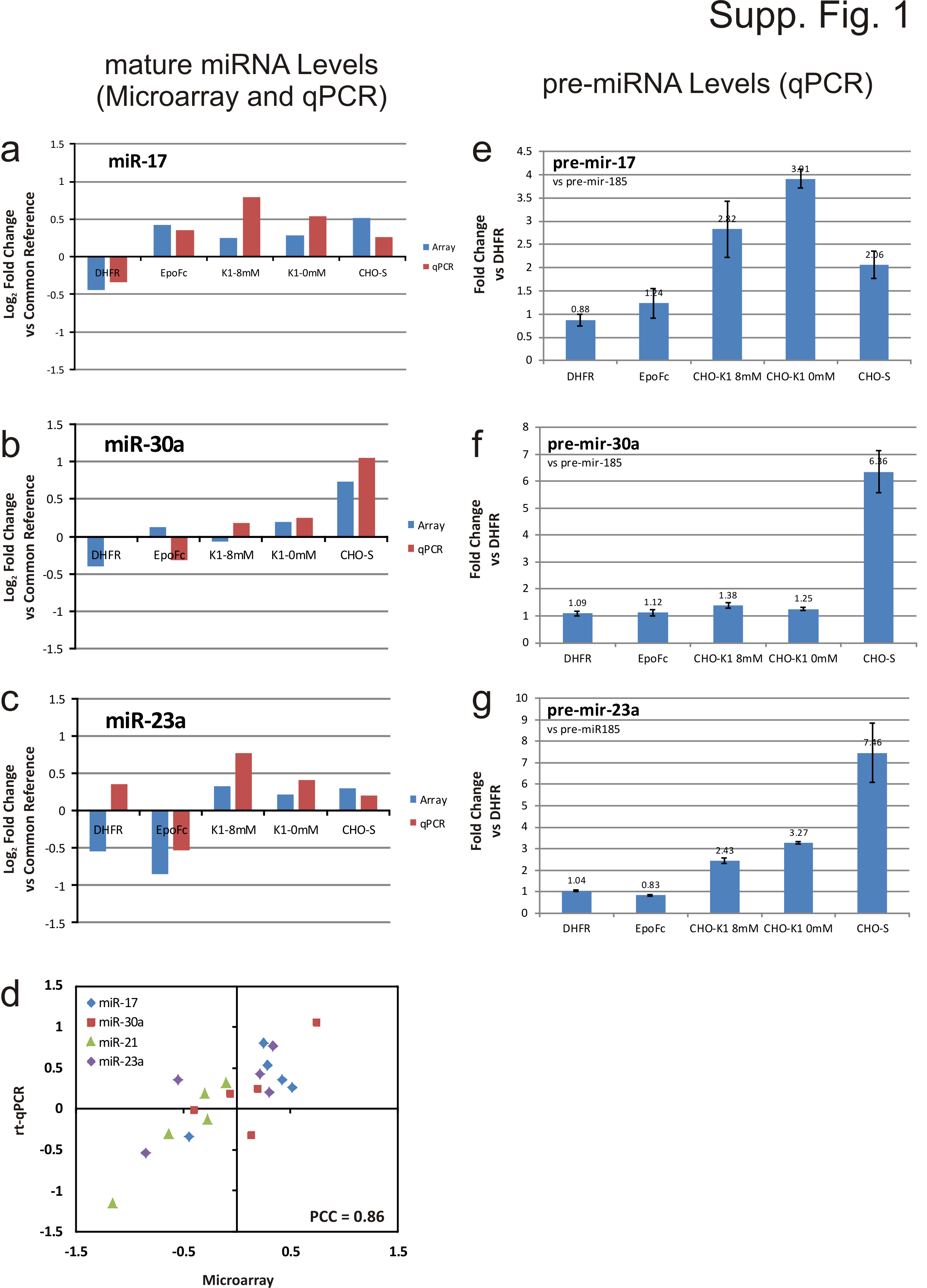


**Supporting Figure 1 – RT-qPCR confirmation of microarray data.** Three miRNAs were selected that showed positive (miR-30a, miR-17, miR-23a) correlation to growth rate after microarray data analysis. Transcript levels for these miRNAs were measured in 5 distinct cell lines in two biological replicates as well as the common reference RNA samples that was used for hybridization. Log_2_ transformed fold changes between cell lines and common reference were derived from microarray and RT-qPCR and overlaid for each miRNA to visualize the agreement of both methods (a-c). (d) Pearson correlation analysis was performed using all 20 data-points. A pearson correlation coefficient of 0.86 was determined. In addition, primers targeting the respective precursor miRNA sequences of miR-30a, miR-17, and miR-23a were designed. Pre-miRNA transcript levels were measured by qPCR in 5 distinct cell lines with 2 replicates each and normalized to pre-mir-185 as reference gene. Fold changes relative to DUKXB-11 (DHFR-) are shown (e-g). Error bars represent RQmin and RQmax.


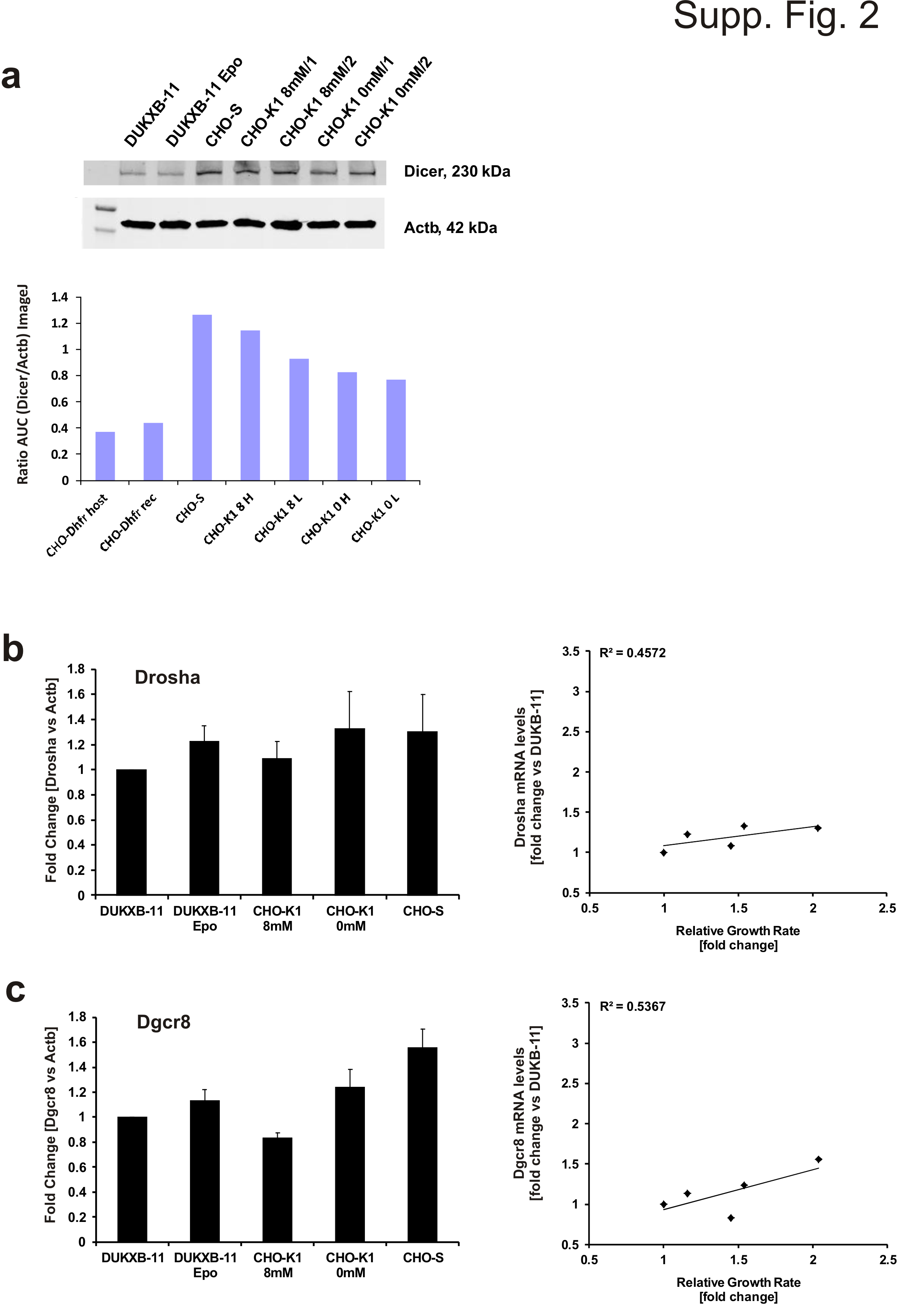


**Supporting Figure 2 – Dicer Immunoblot analysis during exponential growth phase, and Drosha/Dgcr8 correlation to growth rate.** a) Western blot was used to detect Dicer protein in exponential growth phase of 5 CHO cell lines. Actb was used as reference. ImageJ was used to calculate area under the curve (AUC) for Dicer and Actb bands. The ratio of AUC Dicer vs Actb was calculated and is depicted as bar chart. b) Real-time qPCR analysis of Drosha expression (normalized to beta-Actin expression) in 5 protein-free adapted CHO cell lines growing at increasing specific growth rates. Correlation of Drosha expression to growth rate and linear regression of the correlation are shown. c) Analogous analysis for Dgcr8 expression.
